# Talniflumate abrogates mucin immune suppressive barrier improving efficacy of gemcitabine and nab-paclitaxel treatment in pancreatic cancer

**DOI:** 10.1186/s12967-023-04733-z

**Published:** 2023-11-23

**Authors:** Antonio Agostini, Ilaria Guerriero, Geny Piro, Giuseppe Quero, Luca Roberto, Annachiara Esposito, Alessia Caggiano, Lorenzo Priori, Giulia Scaglione, Francesco De Sanctis, Antonella Sistigu, Martina Musella, Alberto Larghi, Gianenrico Rizzatti, Donatella Lucchetti, Sergio Alfieri, Alessandro Sgambato, Emilio Bria, Laura Bizzozero, Sabrina Arena, Stefano Ugel, Vincenzo Corbo, Giampaolo Tortora, Carmine Carbone

**Affiliations:** 1 Medical Oncology, Department of Medical and Surgical Sciences, Fondazione Policlinico Universitario Agostino Gemelli IRCCS, Rome, Italy; 2 Medical Oncology, Department of Translational Medicine and Surgery, Catholic University of the Sacred Heart, Rome, Italy; 3 Biogem, Biology and Molecular Genetics Institute, Ariano Irpino, Italy; 4 Digestive Surgery Unit, Fondazione Policlinico Agostino Gemelli IRCCS, Rome, Italy; 5 Department of Anatomic Pathology, Fondazione Policlinico Universitario Agostino Gemelli IRCCS, Rome, Italy; 6 University Hospital and Department of Medicine, Immunology Section, Verona, Italy; 7 Department of Translational Medicine and Surgery, Università Cattolica del Sacro Cuore, Rome, Italy; 8 Digestive Endoscopy Unit, Fondazione Policlinico A. Gemelli IRCCS, Rome, Italy; 9 Center for Endoscopic Research, Therapeutics and Training, Catholic University of the Sacred Heart, Rome, Italy; 10 General Pathology, Department of Translational Medicine and Surgery, Università Cattolica del Sacro Cuore, Rome, Italy; 11 General Pathology, Department of Medical and Surgical Sciences, Fondazione Policlinico Universitario Agostino Gemelli IRCCS, Rome, Italy; 12 Candiolo Cancer Institute, FPO-IRCCS, Candiolo, TO Italy; 13 Department of Oncology, University of Torino, Candiolo, TO Italy; 14 Department of Engineering for Innovation Medicine (DIMI), University of Verona, Verona, Italy

**Keywords:** Intraductal mucinous neoplasms (IPMNs), Spatial transcriptomics, Pancreatic ductal adenocarcinoma (PDAC), Organoid interaction platform, Syngeneic mouse models

## Abstract

**Background:**

Pancreatic ductal adenocarcinoma (PDAC) is a lethal disease. This is due to its aggressive course, late diagnosis and its intrinsic drugs resistance. The complexity of the tumor, in terms of cell components and heterogeneity, has led to the approval of few therapies with limited efficacy. The study of the early stages of carcinogenesis provides the opportunity for the identification of actionable pathways that underpin therapeutic resistance.

**Methods:**

We analyzed 43 Intraductal papillary mucinous neoplasms (IPMN) (12 Low-grade and 31 High-grade) by Spatial Transcriptomics. Mouse and human pancreatic cancer organoids and T cells interaction platforms were established to test the role of mucins expression on T cells activity. Syngeneic mouse model of PDAC was used to explore the impact of mucins downregulation on standard therapy efficacy.

**Results:**

Spatial transcriptomics showed that mucin *O*-glycosylation pathway is increased in the progression from low-grade to high-grade IPMN. We identified GCNT3, a master regulator of mucins expression, as an actionable target of this pathway by talniflumate. We showed that talniflumate impaired mucins expression increasing T cell activation and recognition using both mouse and human organoid interaction platforms. In vivo experiments showed that talniflumate was able to increase the efficacy of the chemotherapy by boosting immune infiltration.

**Conclusions:**

Finally, we demonstrated that combination of talniflumate, an anti-inflammatory drug, with chemotherapy effectively improves anti-tumor effect in PDAC.

**Supplementary Information:**

The online version contains supplementary material available at 10.1186/s12967-023-04733-z.

## Background

Pancreatic ductal adenocarcinoma (PDAC) is the third leading cause of cancer-related mortality among adults in developed countries, with a median survival of a few months and a 5-year survival less than 8%. It’s considered to become the second cause of cancer death in western societies within a decade [1]. Chemotherapy in most cases is the only treatment for PDAC, while surgery is possible only in a minority of patients (20%). The effectiveness of chemotherapy in treating PDAC depends on several factors, including the stage of diagnosis, patient overall health, and the specific chemotherapy drugs used. The combination of gemcitabine and nab-paclitaxel (Gem/Txl) constitutes the first line therapy in PDAC, however few patients effectively respond to this therapy (< 30%), and many of them have to suspend the therapy for adverse effect caused by its high toxicity (14%) [2]. The implement of non-toxic target therapeutics that enhances Gem/Txl efficacy may markedly improve PDAC therapy.

IPMNs are mucin producing cystic neoplasms that together with pancreatic intraepithelial neoplasia (PanIN), and Mucinous cystic pancreatic neoplasms represent putative precursors of PDAC. The progression from indolent low-grade IPMN to malignant high-grade IPMN, and finally to pancreatic cancer, is associated to local inflammation and failure of protective immunosurveillance [3, 4].

Mucins are high molecular weight glycoproteins found on IPMN membrane and extracellular environment. They form a physical barrier exerting different functions involved in differentiation, adhesion, immune response, and cell signalling [5].

While antitumor immune components, including cytotoxic CD8^+^ T cells, infiltrate the early premalignant stages of IPMN lesions, they are progressively lost during tumor progression to PDAC, accompanied by the concomitant accumulation of immunosuppressive cells [6–8].

The immunosuppressive milieu of pancreatic cancer can be largely attributed to genetic alterations that regulate the tumor microenvironment (TME) [9]. GCNT3 (Glucosaminyl (*N*-Acetyl) Transferase 3) is one of the main enzymes involved in mucin-specific *O*-Glycosylation. In both mouse and human pancreatic cancer tumors, the glycosyltransferase GCNT3 upregulation was correlated with increased expression of mucins [10]. Strategies of immune evasion include also the production of immunosuppressive mucin-type *O*-glycans on mucins that pose a barrier to NKs and T cells [11, 12]. Aberrant GCNT3 expression was associated with increased mucin production, aggressive tumorigenesis, and reduced patient survival, while GCNT3 knock-out in pancreatic cancer cells reduced proliferation and spheroid formation [10, 13]. Talniflumate, a small molecule targeting GCNT3 and developed for cystic fibrosis treatment, can inhibit mucin-specific *O*-glycosylation and expression [14, 15].

In this study, we aimed to identify new actionable targets in PDAC involved in the main biological processes that sustain growth and survival in the early onset. To identify these hallmark pathways we analyzed 43 Intraductal Papillary Mucinous Neoplasm (IPMN) samples including 12 Low-grade (LG) and 31 High-grade (HG) IPMNs performing spatial transcriptomics (GeoMx Human Whole Transcriptome Atlas). We identified mucin-specific *O*-Glycosylation as one of the top tier gene signature differentially regulated between LG and HG IPMNs and along transformation of IPMN to invasive carcinoma.

Taking advantage of in vitro interaction platforms of pancreatic cancer organoids and T cells and of in vivo experiments, we demonstrated that the inhibition of GCNT3 by talniflumate, impairs mucin production, enhances T cells activity and could be used in combination with Gem/Txl to improve chemotherapy efficacy.

These results further highlight the role of mucins in IPMN progression to PDAC by preventing T cell recognition and activation. Furthermore, we demonstrated with an in vivo experiment that GCNT3 is an actionable target in PDAC, paving the way for the development of new strategies targeting the protective mucin barrier and improving the efficacy of PDAC standard chemotherapy.

## Methods

### Patient material

Two Tissue Macro Arrays (TMAs) of clinically annotated IPMNs were kindly provided by Australian Pancreatic Cancer Genome Initiative (APCGI). These TMAs were composed of three normal pancreas samples, 26 Low-Grade and 66 High-Grade IPMNs. TMA slides were stored prior to shipping at -80 °C enclosed in a sealed bag with silica gel beads to avoid RNA oxidation and degradation. The experimental protocol was approved by the local ethics committee (Fondazione Policlinico Gemelli IRCCs, Ethical Committee approval Prot. Gen. 3536) and followed EU regulation.

### PDAC mouse cell lines and 3D cultures

Murine PDAC cell lines DT4313, FC1242, and FC1245 were kindly provided by Dr D. Tuveson’s laboratory, at Cold Spring Harbor Laboratory (New York, USA) and Dr P. Cappello’s laboratory, at CeRMS laboratory (Turin, Italy), and passaged with original growth conditions. Cells were daily inspected and routinely tested to be mycoplasma free by PCR assay. We characterized these models in a previous paper [16] for both transcriptome and immune profiling. From these cell lines we also established 3D cultures KC13 (DT4313), KPC06 (FC1242), KPC12 (FC1245). Briefly, after trypsinization, cell lines were resuspended in 50 μl Cultrex UltiMatrix Reduced Growth Factor Basement Membrane Extract (R&D, Minneapolis, USA) at a defined concentration of 3D structure (n = 150) for each dome. 3D cultures were cultured in PancreaCult™ Organoid Growth Mouse Medium (STEMCELL Technologies, UK) and inspected daily. Human patient derived organoids (PDOs) were established from surgically resected PDAC patients at our institution and cultured as described in [17].

### Spatial transcriptomics

Two IPMN TMAs obtained from Australian Pancreatic Cancer Genome Initiative (APCGI) were analyzed by Spatial Transcriptomics using GeoMX Human Whole Transcriptome Atlas (Nanostring, USA) following the provided protocol. TMAs were stained with GeoMX morphology kit to mark neoplastic cells (PanCK), and immune cells (CD45). We selected 60 ROI filtering out the areas with low cellularity (< 100 nuclei) and that were subjected to tissue disruption and/or detachment. In order to obtain only the IPMN cell-specific transcriptome, segmentation was performed to isolate only the PanCK positive area, excluding the CD45^+^ cells (stroma); therefore only the GeoMx probes specific for IPMN cells were collected for library preparation. GeoMx library was sequenced with NovaSeq 6000 at a coverage of 541 million of total reads. Sequencing raw data was uploaded on Illumina BaseSpace hub and processed with Nanostring GeoMx Digital Spatial Profiling with DRAGEN to obtain.dcc files. The files were imported on R with the GeomxTools R package and quality control (QC) was performed using default parameters. Only the 43 IPMN ROI (12 Low-grade and 31 High-grade) that passed the QC were normalized. GeoMx data was transformed in a Seurat object [19] to perform Differential Expression Analysis (DEA) and for data visualization. The genes resulting from DEA were used for a Gene Set Enrichment kmAnalysis with the R package clusterProfiler using the MSigDB database as reference (www.gsea-msigdb.org). The Seurat function AddModuleScore was used to calculate the average expression levels for the Mucin *O*-glycosylation Program (R-HSA-913709).

### Gene expression and survival analyses

Mucin *O*-glycosilation gene set was obtained from MSigDB database and the normalized counts of these genes were used to perform an explorative data analysis with the R libraries FactoMineR and FactoExtra. Gene expression profiles of *GCNT3, MUC1,* and *MUC5AC* were checked on the TCGA/GTEX PAAD database with the R library UCSCXenaShiny. Prognostic significance for these genes was evaluated with Kaplan–Meier plots generated with KM-plotter [20].

### Histological, and immunofluorescence analyses (IF)

Whole 3 µm tissue sections were dewaxed and rehydrated. Hematoxylin and Eosin staining was performed according to standard protocols. Mucicarmine stain was performed with Mucicarmine Stain Kit (ab150677, Abcam) following standard protocol.

We performed multiplex IF analysis by the Opal 6-Plex Detection Kit (Akoya Biosciences) following standard protocol on a series of 19 IPMN and PDAC tissues from 19 patients (3 low-grade IPMN, 12 high-grade IPMN, and 4 PDAC).

The following antibodies were used for IF analyses: PanCK (M3515, Dako) GCNT3 (PA5-24455, Thermo Fisher Scientific), MUC1 (ab109185, Abcam), MUC5AC (ab3649, Abcam), CD4 (ab288724, Abcam), CD8 (ab251597, Abcam), CD68 (MA5-12407, Invitrogen), CD163 (ab93498, Abcam), iNOS (MA5-17139, Invitrogen). Mucin antibodies were chosen to bind protein regions not affected by *O*-glycosylation, to avoid confounding results given by impaired glycosylation.

Before proceeding, optimal staining conditions for each marker were determined using monoplex stained slides from a positive control for each antibody. Multiplex slides images were acquired by Phenoimager Workstation (Akoya Biosciences, US) and processed with QuPath for cell segmentation and positive cell count.

For organoids analyses FFPE preparation was used by the following protocol. Organoids were collected with Cultrex Organoid Harvesting Solution (R&D, Minneapolis, USA) and incubated in 2% PFA for 15 min. After a brief centrifugation, PFA was discarded, and 3D cultures were included in Histogel (Thermo Fisher Scientific, USA) and left on ice for 3 min. The included 3D cultures were then moved in a histology cassette and incubated in 2% PFA overnight at 4 °C. The next day samples were included in Paraffin. The following antibodies were used: GCNT3 (PA5-24455, Invitrogen), MUC1 (ab109185, Abcam), MUC5AC (ab3649, Abcam). Images were acquired with EVOS (Thermo Fisher Scientific, USA).

For syngeneic orthotopic mouse models the following antibodies were used for IF:

PanCK (81703, SCB), GCNT3 (PA5-24455, Invitrogen), MUC1 (ab109185, Abcam), MUC5AC (ab3649, Abcam), CD8 (ab251597, Abcam), CD68 (97778, CellSignaling), iNOS (MA5-17139, Invitrogen). Citrate Buffer (pH = 6) was used for Antigene Retrieval while a solution of 0.25% Triton-100X in PBS was used for permeabilization. Images were acquired with Phenoimager Workstation scanner and processed with QuPath for cell segmentation and positive cell count.

### Organoids establishment

#### Patient derived organoids

Patient derived organoids (PDOs) were established from PDAC patient samples surgically resected at our Institution. Briefly, tumor tissue was minced in small pieces (1mm^3^) and incubated at 37 °C for 1 h in Digestion Medium containing Advanced DMEM F12, GlutaMAX 1X, HEPES 10 mM, Collagenase II 5 mg/ml, Dispase 1 mg/ml, and Primocin 100 μg/ml. Every 20 min tumor samples were mixed trough a wide bore pipette to maximize cell detachment. Enzymatic digestion was blocked with Washing Medium (DMEM F12 Advanced, GlutaMAX 1X, HEPES 10 mM, and Primocin 100 μg/ml) then filtered through a 70 μm Cell Strainer (Falcon, USA). Filtered solution was centrifuged at 290 rcf for 6 min at RT, washed twice with Washing Medium and finally plated in 50 μl/dome in Cultrex UltiMatrix Reduced Growth Factor Basement Membrane Extract (BME001-10, R&D systems), at the concentration of 5000 structures/well. Organoids were cultured in Growth Medium described by Boj et al. [17] and expanded through passaging every 7/10 days. Only late passages (12–17) were used for this study. The experimental protocol was approved by the local ethics committee (Fondazione Policlinico Gemelli IRCCs, Ethical Committee approval Prot. Gen. 3536) and followed EU regulation. All patients gave consent to participate.

#### Mouse organoids

Pancreatic cancer derived grafts (CDGs) were established and characterized as previously described [16, 21, 22]. For this study we generated organoids from 3 well-established CDG 2D cell lines: KC13 from DT4313, KPC06 from FC1242, and KPC12 from FC1245. Briefly, confluent murine 2D models were detached by standard methods and plated in a 60 μl Cultrex dome at a density of 5 million cells per dome, and cultured in PancreaCult Organoid Growth Medium Mouse (Stemcell Technologies, USA). Fresh medium was added thrice weekly and organoids were firstly expanded only after the establishment of complex structures (10 to 14 days). Only late passages (12–17) were used for this study.

#### In vitro proliferation, migration, and inhibition assays

To identify talniflumate dose in our murine models, we plated 1000 cells/well in 96 well plates in growth medium, containing increasing concentration of the drug (10–500 μM). After 48 h MTT assay was used to obtain relative estimates of viable cell number according to manufacturer instruction.

Migration was assessed by wound-assay, briefly DT4313, FC1242, and FC1245 cells were cultured at full confluence, and wound was formed with pipette. Images were captured after the wounding and at 24 h, and analyzed with ImageJ to estimate the healing ratio.

To estimate talniflumate inhibitory effects on GCNT3, MUC1, and MUC5AC expression, murine 3D cultures were plated at a concentration of 150 organoids/dome and cultured in a growth medium with 100 μM talniflumate. After 72 h, 3D cultures were collected and included in paraffin and used for IF as described above.

### Organoids/T cell interaction platform

#### Human T cell isolation and activation

T cells were isolated from peripheral blood of PDAC patients using the whole blood EasySep™ T Cell Isolation Kit (StemCell Technologies, USA) following manufacturer protocol and cultured in 12 wells plate at concentration of 1 × 10^6^ cells/well in 1 mL of T Cell Culture Medium: RPMI, 10% FBS, 1% l-Glutamine, 1% Hepes, 1% Penicillin/Streptomycin (Gibco, USA), 10 ng/ml IL-2, 10 ng/ml IL-7 (R&D, USA). After 4 h of resting in the incubator, human T cells were activated with Dynabeads Human T-Activator CD3/CD28 (Gibco, USA) for 24 h (The Well Bioscience, UK).

#### Mouse TERT specific T-lymphocytes establishment and culture

Polyclonal mTERT198-205T-Lymphocytes were obtained from a mixed-leukocyte peptide culture set up with vaccinated mice splenocytes in presence of 0.1 μM of mTERT198-205 peptide (VGRNFTNL) according to De Sanctis et al. [18]. Cells were maintained by co-culture with irradiated, syngenic splenocytes pulsed with 0.1 µM TERT peptide in complete medium containing 20 IU/ml of recombinant human IL-2 (Miltenyi Biotec, Germany). OVA257-264-specific CTLs derived from OT-1 splenocytes were stimulated once with 1 µM specific OVA (SIINFEKL) peptide in complete medium containing 20 IU/ml of recombinant human IL-2 (Miltenyi Biotec, Germany) were used as control.

##### PBMCs isolation

PBMC were collected and purified by ACK erythrocytes lysis from C57BL/6 mice, washed in Dulbecco’s Phosphate-Buffered Saline (D-PBS) and used for the following interaction platforms.

#### Interaction platform

Murine and human PDAC 3D cultures (500 organoids/well) were plated in Xeno-free Vitrogel ORGANOID-3 (The Well Bioscience,UK) in a 24 wells plate and treated with 100 μM talniflumate for 48 h. Activated T cells or Mouse PBMC were added to 3D cultures. Organoids were stained with caspase-3/7 activity tracer CellEvent™ Caspase-3/7 Green Detection Reagent (GFP) (ThermoFisher Scientific, USA) to detect apoptosis. T cells were stained with vital staining CellTracker™ Red CMPTX Dye (Texas Red) (Invitrogen, USA) and added to 3D cultures. Fluorescence images were acquired with EVOS FL Auto 2 Cell Imaging System (ThermoFisher Scientific) for 48 h post interaction. 20X GFP fluorescence raw images were quantified with ImageJ program.

### Cytofluorometric analysis

Following the organoid interaction platform they were collected, washed in Dulbecco’s Phosphate-Buffered Saline (D-PBS) and stained with fluorescently labelled mAbs directed against murine CD45 PE-Cya5 (15045182, eBioscience), CD8 PB (48008182, eBioscience), CD25 PE (12025182, eBioscience) in a cold D-PBS solution containing 1% FBS (D-PBS-FBS 1%). Samples were incubated in the dark on ice for 30 min and then washed twice with cold D-PBS-FBS 1% solution before the addition of the Live/Dead Fixable Aqua Dead cell stain as per manufacturer’s instructions. Samples were resuspended in 100 μl growth medium and acquired by means of a CytoFLEX (Beckman Counter, USA) cytofluorometer. Data were analysed by using the FlowJo software v10.0.7.

### In vivo experiments

To generate syngeneic orthotopic mouse models, an incision was made in the left abdominal side at the level of the spleen of C57BL/6 mice. KPC06 organoids were suspended in Cultrex and injected into the tail region of the pancreas using insulin syringes (BD micro-fine 28 Gauge). The injection was considered successful if there were no signs of leakage and the Cultrex sphere containing 3D cultures was visible. The wound was sutured with short-term absorbable suture (Vetsuture, France). After 10 days following transplantation, tumor-bearing mice were subjected to high-contrast ultrasound imaging using the Vevo 2100 System with a MS250, 13–24 MHz scan-head (VisualSonics, The Netherlands) and randomly assigned to the experimental group in order to correct the dimension bias. Talniflumate pre-treatment (medicated diet containing 400 ppm of talniflumate) for mice belonging to talniflumate and combination groups started immediately after randomization. After one week of talniflumate pre-treatment, mice were treated according to each group: control group (saline solution), Gem/Txl and combination group (gemcitabine, 100 mg/kg i.p. once a week for 2 weeks; nab-paclitaxel 10 mg/Kg i.p. once a week for 2 weeks). When at least 50% of control group tumors reached 200 mm^3^, all mice were sacrificed, and excised tumor volume was determined by measuring the long and short axis of the tumor by caliper.

### Statistics

All results, when applicable, were expressed as the means ± SD. Statistical analysis was performed using the Prism 5 software program. Wilcoxon signed-rank test was used to compare groups. P values < 0.05 were considered as statistically significant. Tumor volume curves were generated using the Prism 5 software program and analyzed by log rank tests.

*GCNT3, MUC1,* and *MUC5AC* expression was checked on the TCGA/GTEX PAAD database with the R library UCSCXenaShiny. Prognostic significance for these genes was evaluated with Kaplan–Meier plots generated with KM-plotter (https://kmplot.com).

## Results

### Spatial transcriptomics

**Fig. 1:**
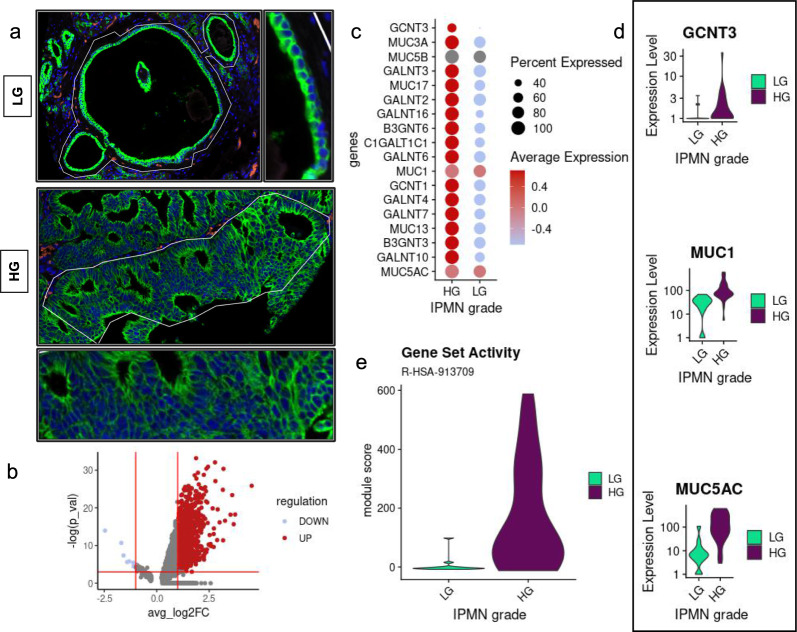
GeoMX spatial transcriptomics analysis. **a** Low-grade (LG), and High-grade (HG) IPMN FPPE samples were stained with PanCk (green) markers for tumor cells, and CD45 (orange) for immune cells detection. Syto13 was used as nuclear staining (blue). **b** GeoMX data was analyzed with Seurat and differential expression analysis was performed for HG compared to LG IPMN. **c**, **d** Mucin genes were consistently overexpressed together with the genes responsible for their *O*-glycosylation. **e** We calculated module score for the mucin *O*-glycosylation signature obtained from Reactome using the addmodulescore() function from Seurat. HG IPMNs showed consistent higher scores in respect to LG, and borderline IPMNs

In order to dissect IPMN transcriptome, we analyzed a total of 43 IPMNs (12 Low-Grade (LG), 31 High-Grade (HG)) for whole spatial transcriptomics (Fig. 1a). To identify pathways deregulated in HG-IPMN in respect to LG-IPMN, we performed DEA (Fig. 1b) followed by GSEA and found that mucin-specific *O*-glycosylation was one of the main pathways enriched in HG-IPMN (Fig. 1c). In keeping with that, both membrane (*MUC1, MUC13*) and secreted (*MUC5AC, MUC5B*) mucins together with genes responsible for their *O*-glycosylation *(GCNT3, B3GNT3, B3GNT6, GALNT2, GALNT3, GALNT4*) were consistently upregulated in HG IPMNs (Fig. 1c). Among those genes, *GCNT3* was consistently overexpressed in HG-IPMN, together with the immune suppressive mucins *MUC1* and *MUC5AC* (Fig. 1d). Moreover, we calculated the gene set activity for the Mucin *O*-glycosylation pathway (R-HSA-913709) using the AddModuleScore function and we found a consistent activation of this pathway in HG IPMNs in respect to LG IPMNs (Fig. 1e) indicating that this process was intensively activated in the malignant IPMN.

### GCNT3, MUC1, and MUC5AC expression in IPMN and PDAC patients

**Fig. 2:**
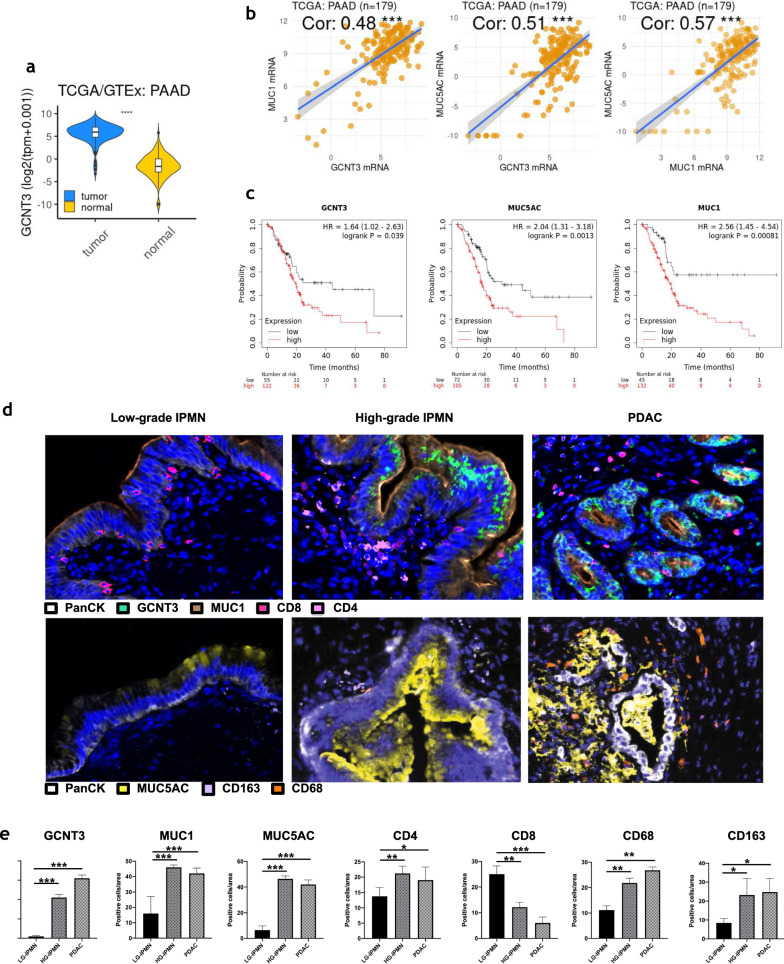
GCNT3, MUC1, and MUC5AC expression in IPMN and PDAC patients. **a** *GCNT3* gene expression values (normalized TPM) from tumor and adjacent normal tissues were reported. T-Student test p < 0.001; **b** Pearson’s *R* correlation coefficient to assess relationships between the mRNA expression levels of *GCNT3*, *MUC1* and *MUC5AC* genes. *P* < 0.05 was considered as statistically significant (*GCNT3* Vs *MUC1*, Cor = 0.48; *GCNT3* Vs *MUC5AC*, Cor = 0.51; *MUC1* Vs *MUC5AC*, Cor 0.57); **c** survival probability curves for TCGA data. Kaplan–Meier survival probability curves for log survival time in the TCGA PDAC data patients. Patients predicted to have long survival times (in black) and for the patients predicted to have short survival times (in red). *GCNT3*, HR = 1.64 (1.02 < CI < 2.63), P = 0.039; *MUC5AC*, HR = 1.64 (1.31 < CI < 3.18), P = 0.0013; *MUC1*, HR = 2.56 (1.45 < CI < 4.54), P = 0.00081; **d** Multiplex IF analysis for PanCK, GCNT3, MUC1, MUC5AC, CD4, CD8, CD163, CD68. Images shown are representative of 1 out of more than 20 fields acquired. **e** Plot showing the counts of positive cell per area (20 ROIs). Kruskal–Wallis test was used to compare differences between sample groups with Low-grade IPMN as control. *** = Pvalue < 0.01, ** = 0.01 > Pvalue < 0.05, * = 0.025 > Pvalue < 0.05. (LG-IPMN) lowgrade IPMN, (HG-IPMN) High-grade IPMN, (PDAC) Pancreatic Ductal Adenocarcinoma

We initially confirmed that *GCNT3* was expressed at higher levels in PDAC tissue respect to normal adjacent tissue in the TCGA PDAC dataset (Fig. 2a). Furthermore, we confirmed that *GCNT3* expression correlates with *MUC1* and *MUC5AC* expression (Pearsons’ coefficient (Cor) 0,48 and 0,51 respectively) (Fig. 2b) and that they were all biomarkers of worse prognosis in pancreatic cancer patients (*GCNT3,* HR = 1.64)*,* (*MUC1,* HR = 2.56), (*MUC5AC*, HR = 2.04) (Fig. 2c). To validate mucin expression in an independent cohort, we assessed the expression of MUC1 and MUC5AC, representing membrane and secreted mucins respectively, in 19 IPMN tissues. Multiplex IF analysis demonstrated that GCNT3 and its related mucins (MUC1, and MUC5AC) were more expressed in PDAC tissues and in the HG-IPMN, in respect to LG-IPMN (Fig. 2d and e). Interestingly, during the progression from low-grade to high-grade IPMN and then to adenocarcinoma (PN0 PN2), and finally to metastasis, the expression of GCNT3 and related mucins appears to increase concomitantly with expression of Epithelial to Mesenchymal Transition (EMT) markers (N-cadherin and Vimentin) (Additional file 1: Figure S1).

Moreover we found, in line with previous studies [6], that low-grade IPMN where characterized by a high number of CD8^+^ T cells whereas the stroma of high-grade IPMN was characterized by the presence of CD68^+^ and CD163^+^ macrophages, and CD4^+^ T cells while CD8^+^ T cells were a minority (Fig. 2d and e).

### Mucin expression in PDAC mouse models

To test the hypothesis that GCNT3 could be a promising therapeutic target, we assessed the effect of talniflumate, a GCNT3 specific inhibitor currently used as anti-inflammatory drug, in PDAC mouse models with different immunogenic potentials that we recently established [16]. Briefly, pancreatic cancer derived graft (CDG) cell lines DT4313, FC1242, FC1245 were characterized by the molecular profile and TME displaying distinct immune features. The DT4313 cell line (classified as classical and immunogenic according to Moffitt and Bailey classification, respectively) has high immunogenic potential. The FC1242 has low immunogenic potential (classified as progenitor and classical according to Moffitt and Bailey classification, respectively) and the FC1245 without any immunogenic potential (classified as basal and ADEX according to Moffitt and Bailey classification, respectively) displays typical features of a cold tumor with absent immune infiltration.

**Fig. 3:**
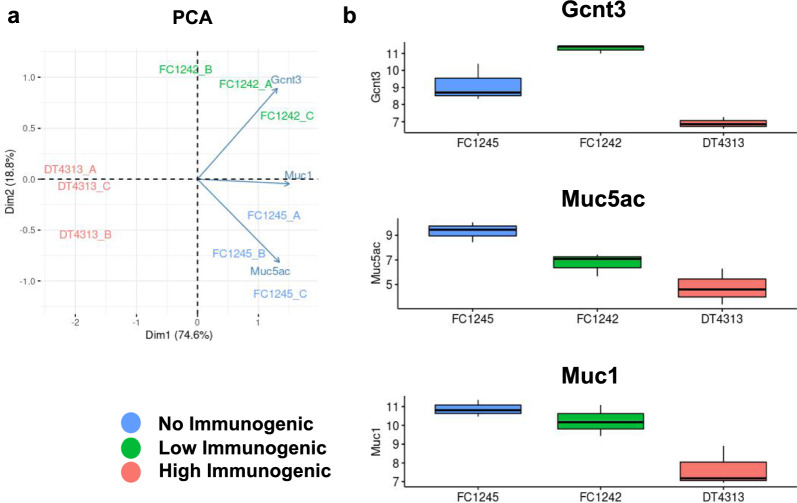
Validation of identified markers in cancer derived graft (CDG) models of pancreatic cancer. **a** PCA plot showing that Mucin-specific *O*-Glycosylation signature distinguish cancer derived grafts (CDG) PDAC murine cell lines DT4313, FC1242, FC1245 analysed for RNAseq. (DIM1, 74.6%, DIM2. 18.8%). Arrows showing Gcnt3, Muc1 and Muc5ac contributions to variance; **b** Gcnt3, Muc1 and Muc5ac expression in CDG cells

**Fig. 4:**
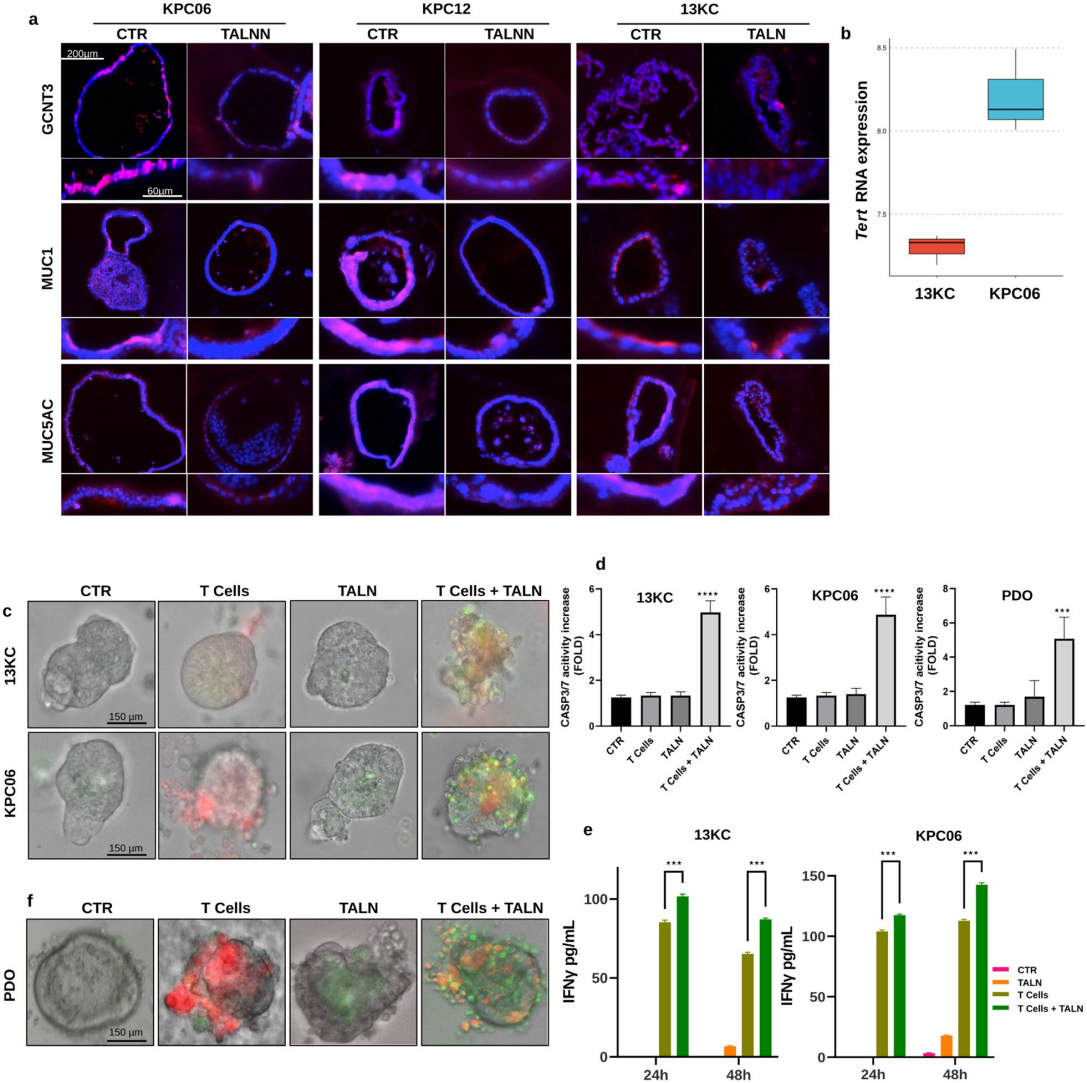
In vitro GCNT3 specific inhibition suppressed mucins deposition promoting T cells infiltration. **a** Immunostaining analysis for GCNT3, MUC1 and MUC5AC. Protein analyzed (in red) and nuclei (in blue) were reported. Images shown are representative of 1–2 out of more than 10 of the 3D cancer cultures acquired. 100X and 200X magnification were reported. **b** Boxplot showing normalized RNA-seq counts for Tert gene in 13KC and KPC06 models. **c** In vitro recognition platform between Telomerase specific T cells and 3D-pancreatic cancer cultures from 13 KC and KPC06 mouse model. T cells are stained with CMPTX (red) and Caspase 3/7 activity is shown in green. **d** In vitro recognition platform between patient derived organoids (PDO) and T cells isolated from the same patient. T cells are stained with CMPTX (red) and Caspase 3/7 activity is shown in green. **e** Barplot showing the fold increase in Caspase 3/7 activity in comparison to control (CTR). The fold increase is calculated as the ratio between the mean of Corrected Total Fluorescence (CTCF) quantified in each group and the mean of control. ****Pvalue < 0.0001, *Pvalue < 0.05. **f** Barplot showing the expression of Interferon γ in pg/ml assessed by ELISA in the conditioned medium from each condition of the interaction platform. ****Pvalue < 0.0001

We assessed the expression of the genes involved in mucins expression in murine models. PCA analysis showed a considerable variance in the expression of genes involved in mucin-specific *O*-glycosylation in our models (Fig. 3a). We found that Gcnt3, Muc1, and Muc5ac, were consistently upregulated in the low (FC1242) or no (FC1245) immunogenic cells respect to the high-immunogenic (DT4313) cell line (Fig. 3b). To study the role of mucins in a more realistic model where these proteins could form a physical 3D barrier able to protect tumors and exclude or suppress immune cells, we established organoid cultures from our CDG model cell lines: KC13 (DT4313), KPC06 (FC1242), and KPC12 (FC1245) (Fig. 4a).

### Talniflumate reduces GCNT3 and mucins expression in organoids

We performed proliferation assay on DT4313, FC1242, and FC1245 models and identified 100 μM as the optimal treatment dose (Additional file 2: Figure S2), in line with previous reports [10]. Moreover the chosen dose was also clinically relevant, as it was less than half the human Cmax [23]. Wound-healing assay on the same cell lines showed no significant difference in migration rate after 100 μM talniflumate treatment at both 24 (Additional file 2: Figure S2) and 48 h.

Immunofluorescence (IF) and western Blot analysis demonstrated that the specific GCNT3 inhibition, by talniflumate, affects mucins and *O*-glcNac expression in the organoid models (Fig. 4a and Additional file 3: Figure S3). The expression of MUC1 and MUC5AC, representing respectively membrane and secreted mucins with immunosuppressive effects, were evaluated. To overcome the possible mismatch caused by impaired glycosylation, we selected antibodies targeting the mucin proteins lacking glycosylation modification sites. IF indicated that talniflumate reduced both GCNT3 and mucins (MUC1 and MUC5AC) expression in all our models (Fig. 4a) hinting that the drug effect on mucin barrier disruption may be imputed to the downregulation of mucin proteins.

To determine if the blockade of GCNT3 and, in turn, the expression and glycosylation of mucins can lead to increased susceptibility of tumor cells to immune system cellular components in terms of recognition and killing we established interaction platforms between KPC06 and 13KC mouse organoids and PBMCs isolated from syngeneic mice (Additional file 4: Figure S4). We demonstrated that talniflumate pre-treatment of organoids increases the recognition of tumor cells by immune cells (Additional file 4: Figure S4a, red), apoptosis (Additional file 4: Figure S4a, green) and T lymphocytes activation (CD8^+^/CD25^+^) (Additional file 4: Figure S4b). The PBMCs in the interaction platforms were used to partially compensate for the absence of lymphoid organs where T-lymphocytes can be committed for the recognition of tumor cells. Therefore, we hypothesized that this result may underestimate the actual efficacy of GCNT3 tumor inhibition.

To evaluate the effect of talniflumate on the activity of T cells in the recognition of cancer cells we established an interaction platform between KPC06 and 13KC mouse organoids and TERT specific T-lymphocytes, as described by De Sanctis et al. [18]. Briefly, we initially evaluated the expression of TERT epitope in our cancer models (Fig. 4b). Then we demonstrated that talniflumate pre-treatment of 13 KC and KPC06 increases the recognition of tumor cells by immune cells (Fig. 4c, red), the apoptosis by caspase activity monitoring in tumor cells (Fig. 4c, green, and d) and T lymphocytes activation (interferon γ release) (Fig. 4e). Moreover, to confer a clinical relevance of this study we investigated talniflumate activity also in a patient derived organoid platform (Fig. 4f). For this specific organoid-immune interaction platform, T lymphocytes were collected and purified from the peripheral blood of the same patient, then expanded and activated in vitro. As expected activated T cells were able to recognize and kill organoids only if these latter were pre-treated with talniflumate.

These data indicate that GCNT3 participates in the formation of mucin physical barrier surrounding cancer cells and that its inhibition disrupts this barrier allowing tumor recognition by T cells.

### Talniflumate reduces GCNT3 and mucins expression in in vivo experiments

To test in vivo the effects of talniflumate, we orthotopically transplanted KPC06 organoid models into immunocompetent and syngeneic mice (n = 20). Tumor bearing mice were randomly assigned to 4 groups (n = 5) to receive saline as control (CTR), talniflumate diet regimen (TALN), or a human standard therapy gemcitabine/nab-paclitaxel (Gem/Txl) alone or in combination with the talniflumate diet regimen (COMB).

**Fig. 5:**
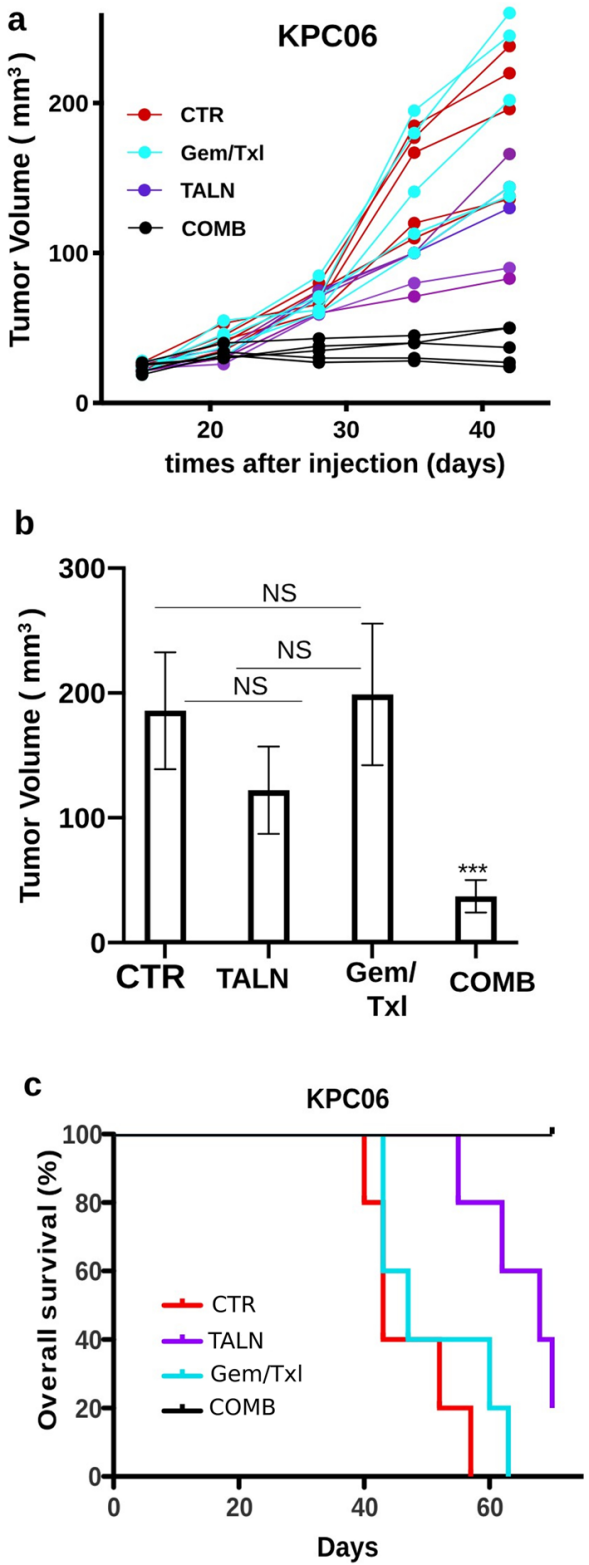
GCNT3 inhibition enhanced Gem/Txl standard therapy efficacy in orthotopic syngeneic PDAC mouse model. **a** Plot showing individual tumor growth curves of KPC06 tumor bearing mice (n = 5) randomly assigned to receive saline (CTR), standard therapy (Gem/Txl), talniflumate (TALN) and talniflumate-Gem/Txl combination treatment (COMB). **b** Barplot showing tumor growth grouped by treatment. Wilcoxon signed-rank test was used to compare differences between treatments. *** = Pvalue < 0.01, * = Pvalue < 0.05, NS = not significant. **c** Plot showing the overall survival of KPC mice divided according each experimental condition

**Fig. 6:**
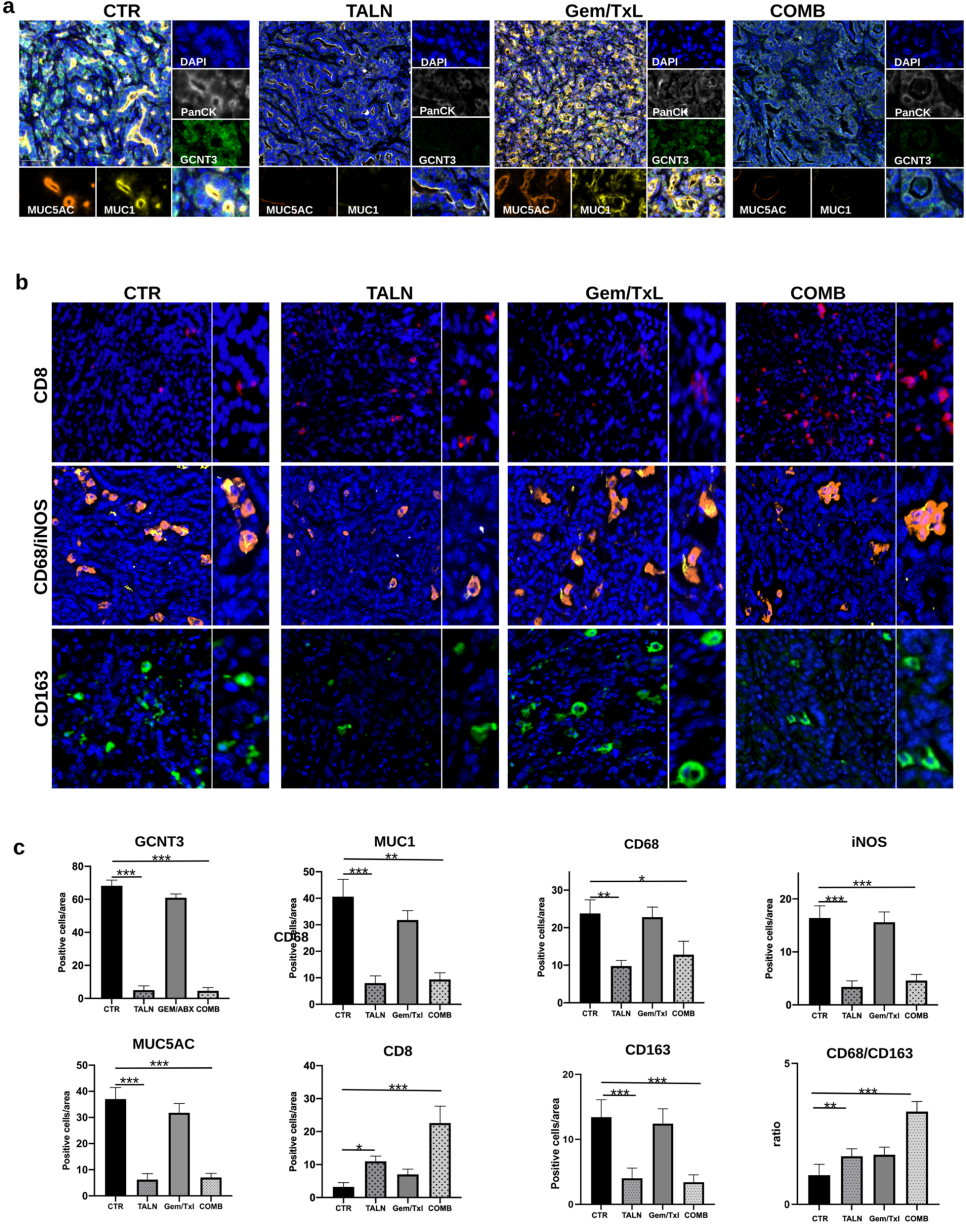
Immunofluorescence analysis on orthotopic syngeneic PDAC mouse model. **a** Multiplex IF analysis showing the correlation of PanCK, GCNT3, MUC1, MUC5AC in KPC06 mice models randomly assigned to receive saline (CTR), standard therapy (Gem/Txl), talniflumate (TALN) and talniflumate-Gem/Txl combination treatment (COMB). Images shown are representative of 1 out of more than 10 fields acquired and reviewed by pathologist. **b** IF showing the expression of CD8, CD68, iNOS, and CD163 in the same experimental groups. Images shown are representative of 1 out of more than 10 fields acquired and reviewed by pathologist. **c** Barplots show percentage of positive cells per area grouped by treatments. Kruskal–Wallis test was used to compare differences between treatments and control. **** = Pvalue < 0.0001, *** = Pvalue < 0.001, *** = Pvalue < 0.01,* = Pvalue < 0.05

As anticipated, the tumors experienced a limited response to the common chemotherapy treatment regimen (Gem/Txl) (Fig. 5a and b). The incorporation of talniflumate alongside the standard therapy markedly decreased tumor progression (p < 0.01) (Fig. 5a and b), leading to an extension in overall survival (Fig. 5c). IF analyses (Fig. 6a and c) and Mucicarmine stain (Additional file 5: Figure S5) of the excised tumors demonstrated that talniflumate reduced GCNT3 and mucins expression also in vivo*.*

The immune system balance of macrophages and CD8 positive T lymphocytes was affected by talniflumate with a statistical significance. Specifically, all macrophages appear to decrease, with a greater percentage in pro-tumoral M2 CD163^+^ macrophages compared to M1 CD68^+^/iNOS^+^ macrophages (Fig. 6b and c). As expected from data already obtained in human and murine platforms, a reduction in the levels of MUC1 and MUC5AC expression leads to a higher accumulation of CD8^+^ T lymphocytes (Fig. 6b and c), which could explain the shrinkage of tumors following treatment with talniflumate.

All these results showed that talniflumate is able to evoke an anti-tumoral immune response in syngeneic PDAC mouse models, however only the combination treatment of talniflumate with standard therapy was able to concomitantly increase the number CD8^+^ T cells infiltrating the tumors and produce a significant tumor volume shrinkage (Figs. 5a, b and 6a–c). Overall, this data indicates GCNT3 as a promising therapeutic target and the administration of talniflumate in combination with standard treatment as a possible therapeutic option.

## Discussion

Pancreatic cancer is a deadly disease with few therapeutic options and overall poor efficacy. Gemcitabine is often used as a single agent or in combination with other chemotherapy drugs like nab-paclitaxel (protein-bound Paclitaxel), to treat advanced disease [2]. Several studies showed the poor efficacy of Gem/Txl with only around 23% of overall response rate [24].

IPMN are pancreatic cysts with malignant potential, deriving from epithelium of the main or collateral branch ducts. The progression of this lesion from nonmalignant Low-grade IPMN to High-grade IPMN and finally PDAC is marked by the acquisition of distinct molecular and histological features [4]. Moreover, Bernard et al. [6] showed by single-cell RNA sequencing that Low-grade IPMN possess a pro-inflammatory stroma that is gradually lost during the degeneration to PDAC. The prevalence of these cysts is currently in constant increase reaching around 8% of the global population [25]. Thus, the identification of earliest molecular events responsible for PDAC onset remains critical not only for early detection and prevention, but also to provide an opportunity for the targeting of novel potential vulnerabilities.

In this study we focused in the cancer cell-specific mechanisms that drive IPMN progression. Among the top activated gene signatures identified by our Spatial Transcriptomic investigation, we focused our attention on the “Mucin-specific *O*-Glycosylation” gene signature. In particular we found that *GCNT3*, an essential enzyme in mucin biogenesis, was a specific marker of IPMN malignancy (Figs. 1c, 2d, e and Additional file 1: Figure S1).

Several papers reported the role of mucins in forming the physical barrier that protects normal epithelia from injury [26]. It has been reported an aberrant expression of mucins in many types of disease including solid tumors [27] and that mucins could modulate directly immune response [11, 28–32]. It has also been demonstrated that mucin-specific *O*-Glycosylation influences a broad range of signalling pathways to promote disease onset and progression sustaining proliferative and pro-tumorigenic signalling, contributing to pancreatic cancer phenotypes [33, 34] and facilitating tumor immune escape [11, 35]. In particular, aberrant mucin glycosylation on cancer cells leads to expression of atypical epitope resulting in a specific recognition and binding of cancer cell membrane glycosylation patterns, leading to apoptosis of cancer specific effector T cells [36]. Aberrant expression of Glycosyltransferase enzyme GCNT3, a master regulator of mucin-specific *O*-Glycosylation, was associated with increased production of mucins, reduced patient survival and chemoresistance in colon and ovarian cancer [37]. Specific inhibition of GCNT3 by talniflumate, an orally available, small-molecule inhibitor and a muco-regulator, blocks mucous overproduction in patients with chronic respiratory diseases, and reduces cystic fibrosis [14]. Moreover, it has been reported its activity in blocking pancreatic cancer cell proliferation and pancreatic intraepithelial neoplasia (PanIN) in vivo formation, mainly by downregulation of mucins expression [13]. Gupta and colleagues [10] also showed that talniflumate inhibits PDAC cell lines proliferation by downregulation of mucins and inhibition of their cell-intrinsic functions. On the basis of these promising results, we wanted to evaluate the effect on cancer immunity of talniflumate-mediated mucin suppression using organoids as models for both in vitro*,* and in vivo experiments. To test the immune suppressive role of mucin barrier in a realistic model and to address the role of GCNT3 and mucins in immune escape, we established an in vitro recognition platform to track the cellular interactions between cancer and T cells. The organoid platform experiments, with both mouse and human 3D PDAC models, demonstrated that talniflumate abrogates mucins secretion facilitating T cells functions and highlighting the strong effect of this small molecule on immune modulation (Fig. 4 and Additional file 4: Figure S4). We confirmed these results in an orthotopic syngeneic mouse model with low immunogenic potential, in which talniflumate enhanced Gem/Txl efficacy provoking a significant reduction in tumor growth (Fig. 5a and b) and increas in overall survival (Fig. 5c). IF analyses suggested that the main effect of talniflumate was an increase of T cell infiltration into the tumor (Fig. 6b and c). However, these are proof of concept findings and further studies are needed for clinical applicability. Randomized clinical trials with the administration of talniflumate in addiction or not to standard chemotherapy could definitively demonstrate the efficacy of *O*-glycosylation inhibition on chemotherapy response and finally on patient outcome. This translational path can guide future efforts to successfully translate our findings into healthcare.

## Conclusions

The development on novel target therapies in advanced PDAC is an unmet need. In this work we used spatial transcriptomics to understand the key processes activated along PDAC development studying one of the most comprehensive samples series analyzed with this technology so far. We identified GCNT3 as a marker of IPMN transformation into invasive carcinoma and a suitable therapeutic target for pancreatic cancer treatment. We showed that GCNT3 expression increases in High-grade IPMN (Fig. 2d) and is conserved in PDAC and metastasis (Additional file 1: Figure S1) where it defines a population with an adverse prognosis (122 patients out of 177, TCGA data) (Fig. 2c), entailing its eligibility as target for a novel therapy.

**Fig. 7:**
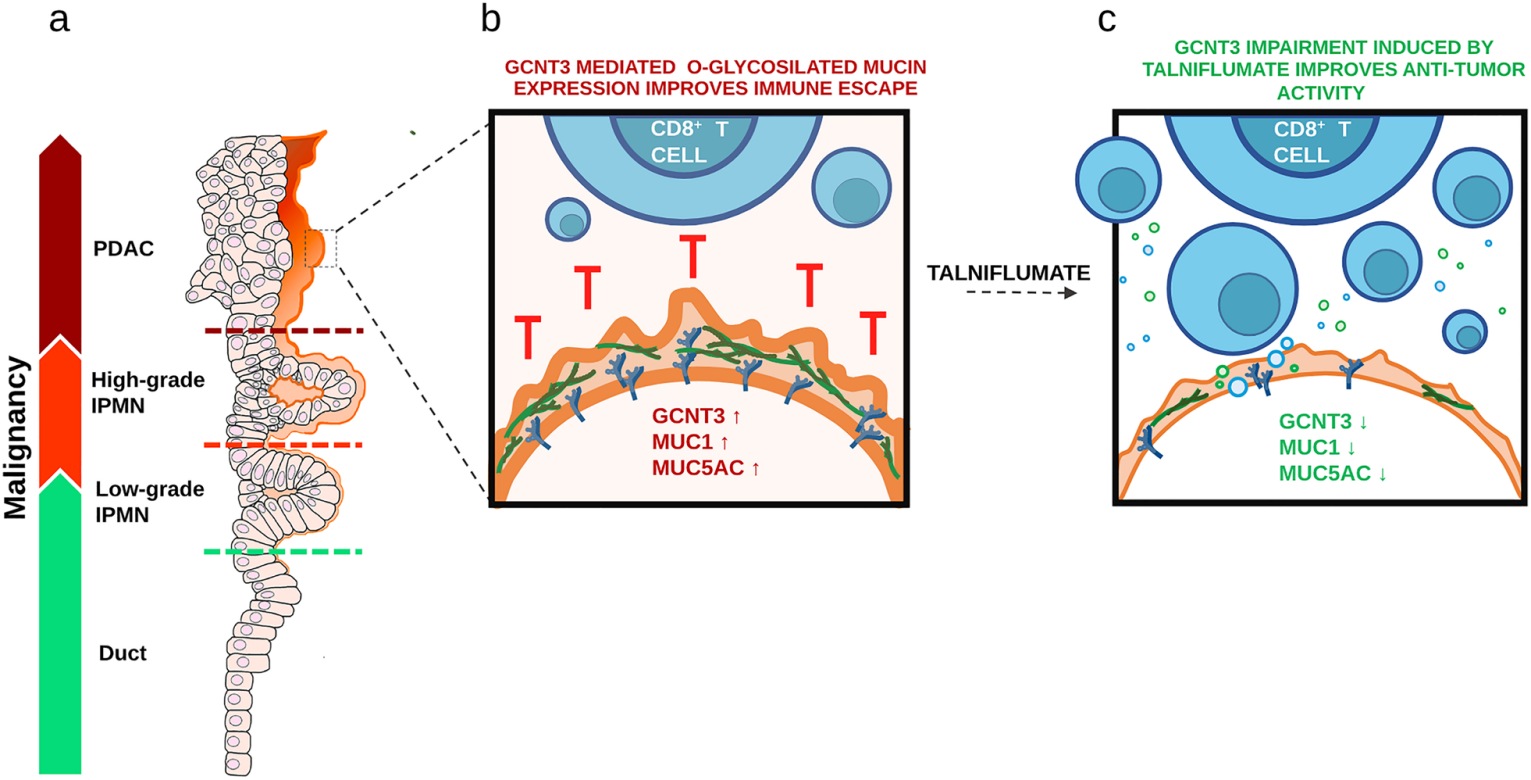
Graphical summary of the study main results. **a**) GCNT3 and Mucin expression increase throughout the IPMN from Low-grade IPMN to High-grade IPMN and PDAC. **b** *O*-glycosilated Mucins shield the tumor cells from T cells improving immune escape. **c** Talniflumate abrogates GCNT3 activity and impairs teh enzyme expression together with MUC1 and MUC5AC favoring T cell recognition and activation

We validated the efficacy of GCNT3 inhibition by talniflumate in both in vitro and in vivo experiments on both human and mouse organoids. We showed that GCNT3 inhibition by talniflumate disrupts the expression of both membrane and secreted mucins impairing the formation of an immune suppressive barrier that protects cancer cells from T cells. Talniflumate boosts immune infiltration into the tumor core and significantly improves Gem/Txl therapy paving the way to a novel approach for treating PDAC with immediate impact in the clinical setting (Fig. 7).

### Supplementary Information

**Additional file 1: Figure S1.** GCNT3, Vimentin, and N-cadherin expression throughout PDAC progression. a) Multiplex IF showing the expression of GCNT3, Vimentin, and N-cadherin in Low-grade IPMN, High-grade IPMN, locally advanced PDAC (pN0), metastatic PDAC (pN2) and matched metastasis. b) Barplots show percentage of positive cells per area in the 10 different ROI annotated by pathologist.

**Additional file 2: Figure S2.** In vitro proliferation and migration assays. a) Plot showing proliferation curves for DT4313, FC1242, FC1245 treated with increasing concentration of talniflumate. b) Images showing Wound-Healing assay at T0 and after 24 h in DT4313, FC1242, FC1245 cell lines. c) Barplot showing the healing ratio after 24 h for control and treated (talniflumate 100 μM).

**Additional file 3: Figure S3.** Talniflumate decrease the expression of GCNT3, MUC1, MUC5AC, and total *O*-glycosilation. a) Western-blot shows the effect of talniflumate (100 μM) GCNT3, MUC1, and MUC5AC in DT4313, FC1242, FC1245 models. b) Western blot with anti-*O*-GlcNAc show decrease of total *O*-glycosilation in n DT4313, FC1242, FC1245 models treated with 100 μM talniflumate.

**Additional file 4: Figure S4.** Syngeneic interaction platform between PBMC and CDG mouse models. a) In vitro recognition platform between PBMC and 3D-pancreatic cancer cultures from KPC06 mouse model. PBMC are stained with CMPTX (red) and Caspase 3/7 activity is shown in green. b) Barplot showing the fold increase in Caspase 3/7 activity in comparison to control (CTR). The fold increase is calculated as the ratio between the mean of Corrected Total Fluorescence (CTCF) quantified in each group and the mean of control. * pvalue < 0.05. c) Barplot showing the fold increase CD8 + /CD25 + T cells in comparison to control (PBMC) assessed by cytometry.

**Additional file 5: Figure S5.** Histochemical assays on orthotopic syngeneic PDAC mouse model. Figure panel showing Hematoxylin and Eosin (H&E) and Mucicarmine stains on orthotopic syngeneic PDAC mouse model. Images shown (20X) are representative of 1 out of more than 10 fields acquired and reviewed by pathologist. (CTR), standard therapy (Gem/Txl), talniflumate (TALN) and talniflumate-Gem/Txl combination treatment (COMB).

## Data Availability

Spatial transcriptomics data is avaliable on GEO database.

## Abbreviations

IPMN: Intraductal mucinous neoplasm
PDAC: Pancreatic ductal adenocarcinoma
HG: High-grade dysplasia
LG: Low-grade dysplasia
ROI: Region of interest
PCA: Principal component analysis
FFPE: Formalin Fixed Paraffin Embedded
CDG: Cancer derived grafts
TCGA: The Cancer Genome Atlas
IF: Immunofluorescence
IHC: Immunohistochemistry
Cor: Pearsons’ coefficient
HR: Hazard Ratio
CTR: Control
TALN: Talniflumate
Gem/Txl: Gemcitabine/Abraxane
COMB: Combination
NK: Natural killer
PanIN: Pancreatic intraepithelial neoplasia
