# Supplementary material for: Talniflumate abrogates mucin immune suppressive barrier improving efficacy of gemcitabine and nab-paclitaxel treatment in pancreatic cancer
Source: J Transl Med. 2023 Nov 23;21:843. doi: 10.1186/s12967-023-04733-z (PMC10668479; doi:10.1186/s12967-023-04733-z)
Supplement: Supplementary file 1 — Additional file 1: Figure S1. GCNT3, Vimentin, and N-cadherin expression throughout PDAC progression. a) Multiplex IF showing the expression of GCNT3, Vimentin, and N-cadherin in Low-grade IPMN, High-grade IPMN, locally advanced PDAC (pN0), metastatic PDAC (pN2) and matched metastasis. b) Barplots show percentage of positive cells per area in the 10 different ROI annotated by pathologist. [file 12967_2023_4733_MOESM1_ESM.pdf]

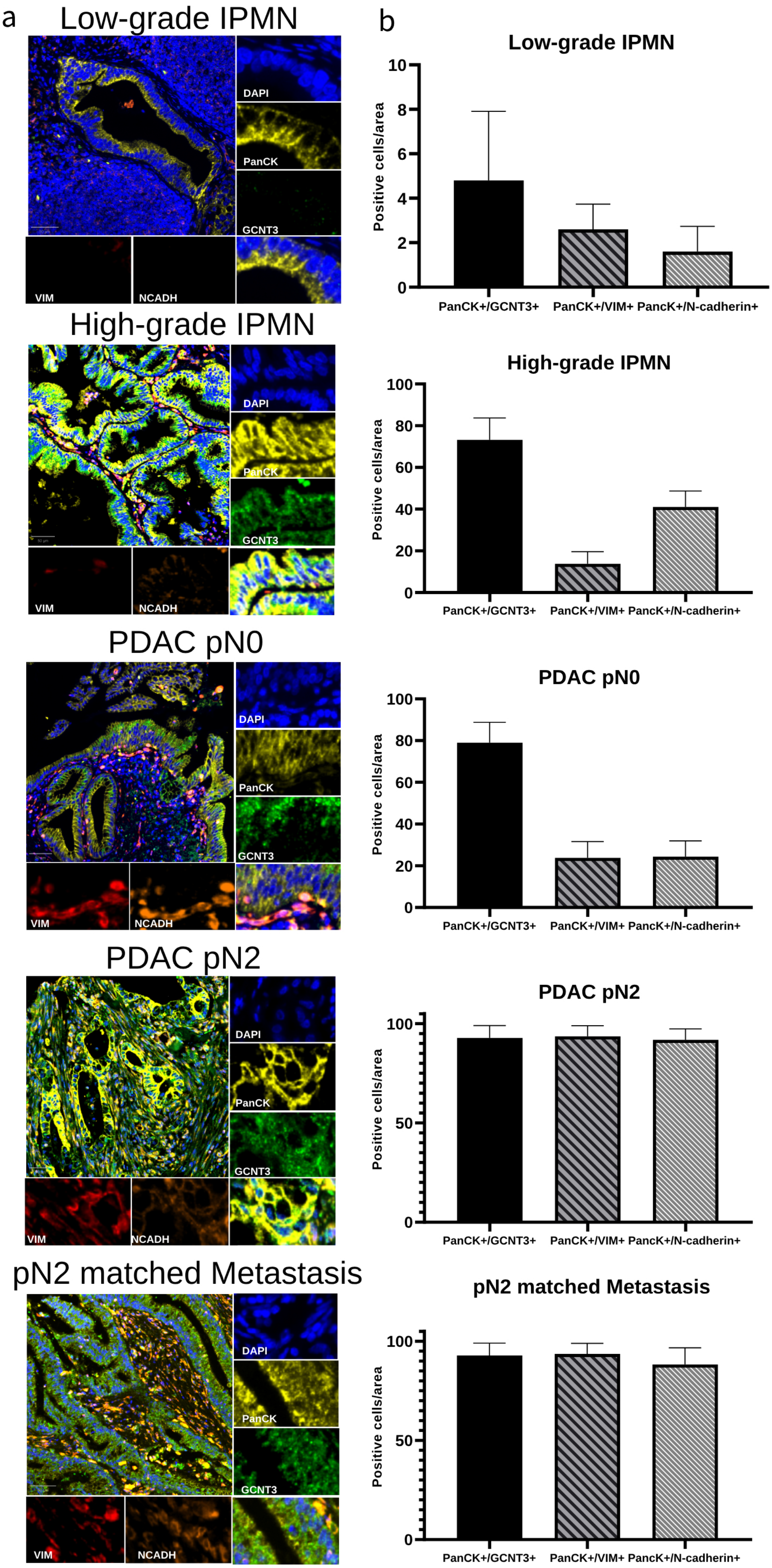
a
Low-grade IPMN
DAPI
PanCK
GCNT3
VIM
NCADH
High-grade IPMN
DAPI
PanCK
GCNT3
VIM
NCADH
PDAC pN0
DAPI
PanCK
GCNT3
VIM
NCADH
PDAC pN2
DAPI
PanCK
GCNT3
VIM
NCADH
pN2 matched Metastasis
DAPI
PanCK
GCNT3
VIM
NCADH
b
Low-grade IPMN
Positive cells/area
PanCK+/GCNT3+
PanCK+/VIM+
PanCK+/N-cadherin+
High-grade IPMN
PDAC pN0
PDAC pN2
pN2 matched Metastasis
