# Supplementary figures and images for: Talniflumate abrogates mucin immune suppressive barrier improving efficacy of gemcitabine and nab-paclitaxel treatment in pancreatic cancer
Source: J Transl Med. 2023 Nov 23;21:843. doi: 10.1186/s12967-023-04733-z (PMC10668479; doi:10.1186/s12967-023-04733-z)

a

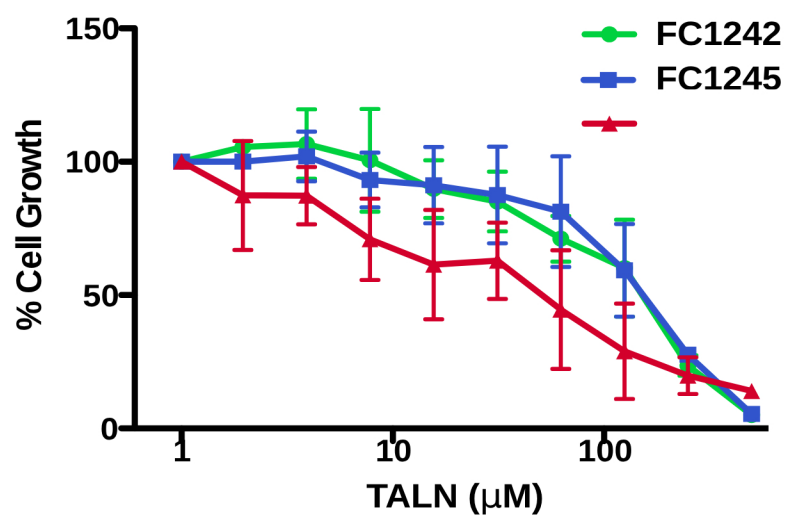

b

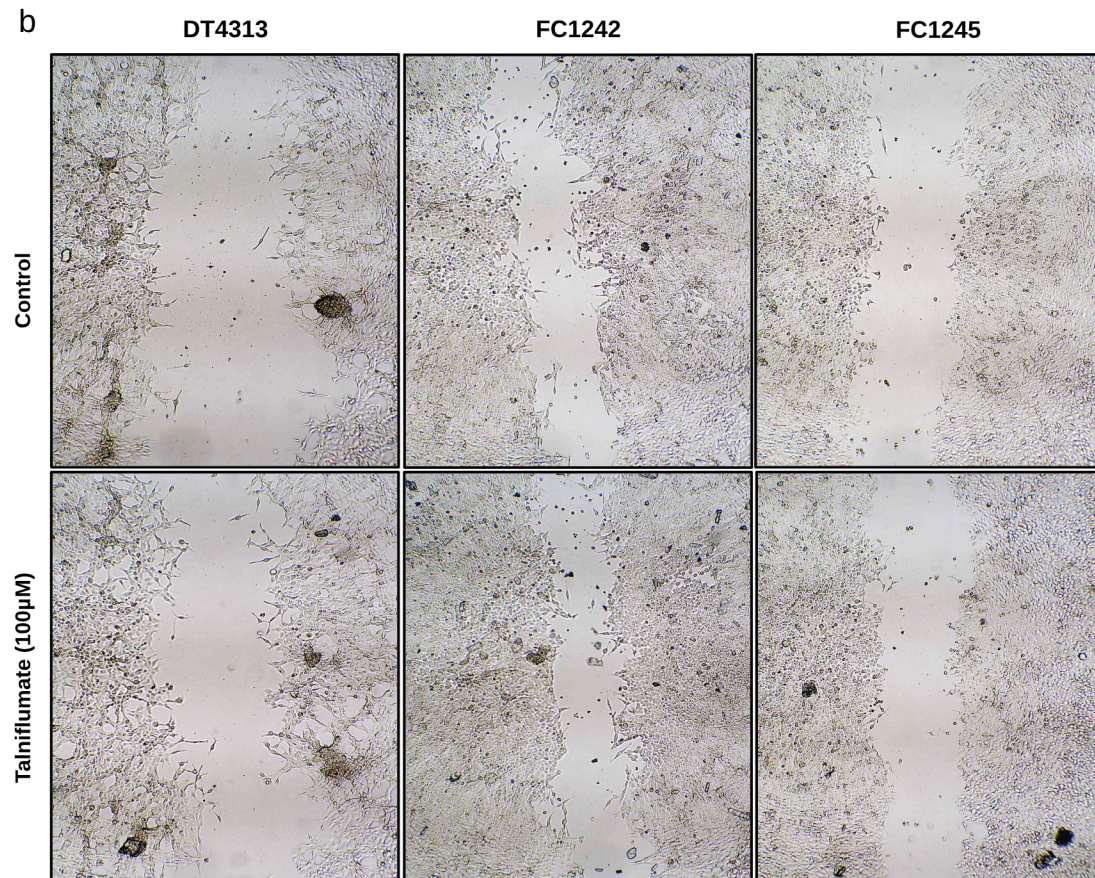

c

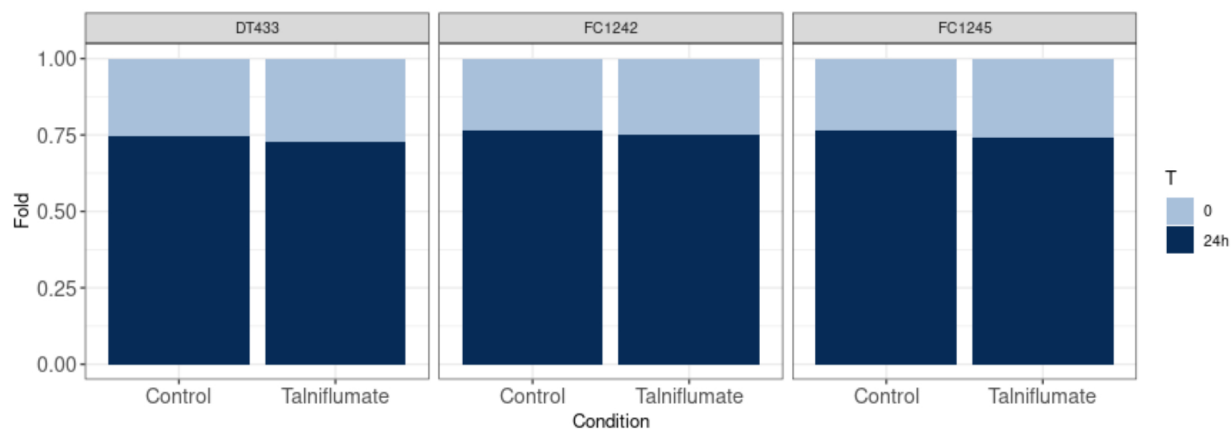

Supplement: Supplementary file 2 — Additional file 2: Figure S2. In vitro proliferation and migration assays. a) Plot showing proliferation curves for DT4313, FC1242, FC1245 treated with increasing concentration of talniflumate. b) Images showing Wound-Healing assay at T0 and after 24 h in DT4313, FC1242, FC1245 cell lines. c) Barplot showing the healing ratio after 24 h for control and treated (talniflumate 100 μM). [file 12967_2023_4733_MOESM2_ESM.pdf]

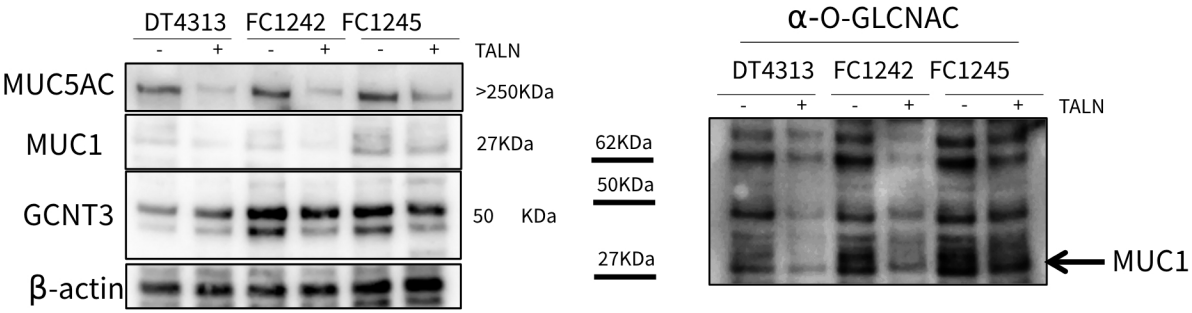

Supplement: Supplementary file 3 — Additional file 3: Figure S3. Talniflumate decrease the expression of GCNT3, MUC1, MUC5AC, and total O-glycosilation. a) Western-blot shows the effect of talniflumate (100 μM) GCNT3, MUC1, and MUC5AC in DT4313, FC1242, FC1245 models. b) Western blot with anti-O-GlcNAc show decrease of total O-glycosilation in n DT4313, FC1242, FC1245 models treated with 100 μM talniflumate. [file 12967_2023_4733_MOESM3_ESM.pdf]

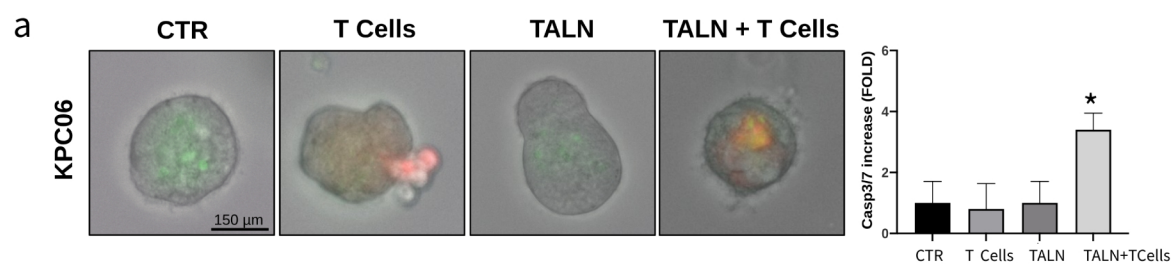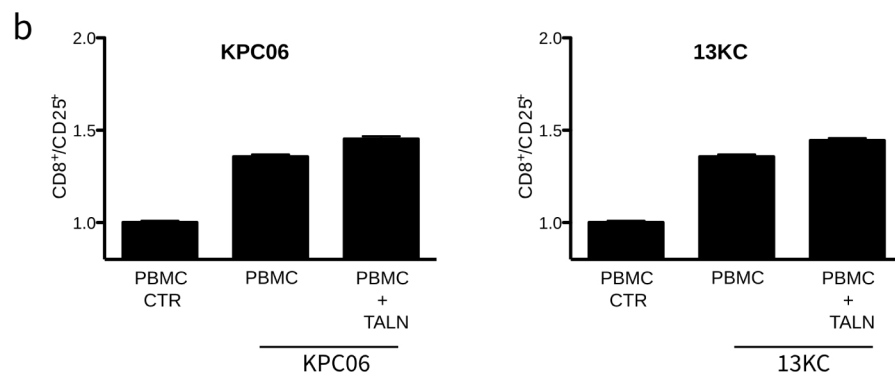

Supplement: Supplementary file 4 — Additional file 4: Figure S4. Syngeneic interaction platform between PBMC and CDG mouse models. a) In vitro recognition platform between PBMC and 3D-pancreatic cancer cultures from KPC06 mouse model. PBMC are stained with CMPTX (red) and Caspase 3/7 activity is shown in green. b) Barplot showing the fold increase in Caspase 3/7 activity in comparison to control (CTR). The fold increase is calculated as the ratio between the mean of Corrected Total Fluorescence (CTCF) quantified in each group and the mean of control. * pvalue < 0.05. c) Barplot showing the fold increase CD8 + /CD25 + T cells in comparison to control (PBMC) assessed by cytometry. [file 12967_2023_4733_MOESM4_ESM.pdf]

CTR

TALN

Gem/Txl

COMBO

H&E

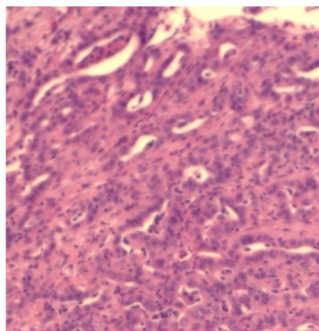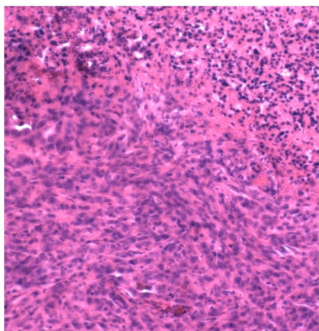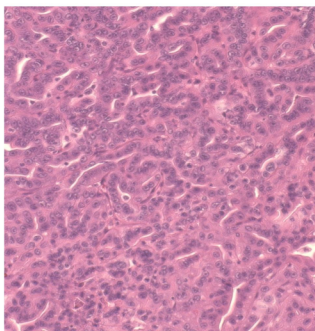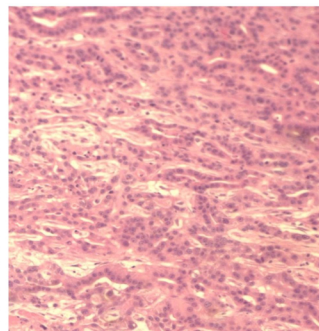

Mucicarmine

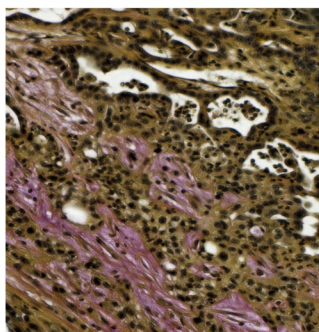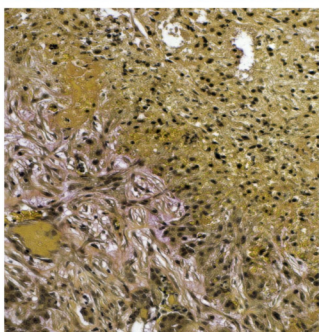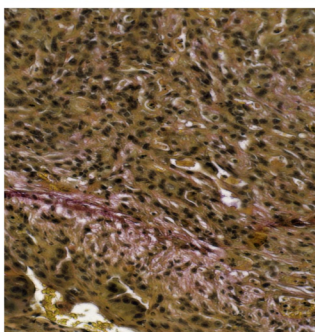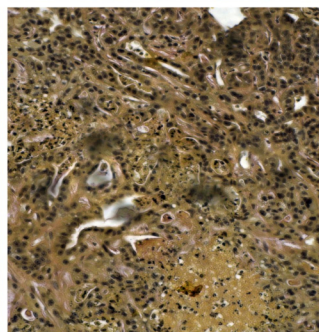

Supplement: Supplementary file 5 — Additional file 5: Figure S5. Histochemical assays on orthotopic syngeneic PDAC mouse model. Figure panel showing Hematoxylin and Eosin (H&E) and Mucicarmine stains on orthotopic syngeneic PDAC mouse model. Images shown (20X) are representative of 1 out of more than 10 fields acquired and reviewed by pathologist. (CTR), standard therapy (Gem/Txl), talniflumate (TALN) and talniflumate-Gem/Txl combination treatment (COMB). [file 12967_2023_4733_MOESM5_ESM.pdf]
